# Chondroblastic Osteosarcoma of proximal tibia

**DOI:** 10.4322/acr.2024.515

**Published:** 2024-09-12

**Authors:** Ravi Hari Phulware, Kaashvi Gupta, Gaurav Rajendra Shirsath, Mohit Dhingra

**Affiliations:** 1 All India Institute of Medical Sciences, Department of Pathology & Laboratory Medicine, Rishikesh, Uttarakhand, India; 2 All India Institute of Medical Sciences, Department of Orthopedics, Rishikesh, Uttarakhand, India

**Keywords:** Osteosarcoma, Chondrocytes, Tibia, Chondroblastic, Osteoid

Chondroblastic osteosarcoma is a unique form of osteosarcoma defined by malignant cells producing osteoid and cartilaginous matrix.^1,2^ While osteosarcoma is an uncommon disease, accounting for less than 1% of all cancers, chondroblastic osteosarcoma accounts for 25-30% of osteosarcoma occurrences.^2,3^ It commonly affects the metaphyseal areas of long bones, particularly the distal femur, proximal tibia, and proximal humerus.^2^ This discussion aims to evaluate the existing literature on chondroblastic osteosarcoma of the upper tibia, focusing on significant findings, diagnostic problems, treatment methods, and results.

Patients with chondroblastic osteosarcoma of the upper tibia typically present with nonspecific symptoms such as localized pain, swelling, and restricted range of motion.^3,4^ In some circumstances, pathological fractures can occur, causing symptoms to worsen suddenly. However, due to the deep-seated nature of the tumor within the bone, clinical discovery may be delayed, resulting in advanced disease at diagnosis.^1,3^

Chondroblastic osteosarcoma is diagnosed based on clinical, radiographic, and histological findings. Radiographically, the tumor typically appears as an aggressive lytic lesion with areas of mineralization and cortical damage.^4,5^ However, identifying chondroblastic osteosarcoma from other bone tumors, such as chondrosarcoma or Ewing sarcoma, can be difficult based only on imaging. Histopathological analysis of a biopsy specimen is required for a definitive diagnosis, which reveals the presence of osteoid and cartilaginous matrix generated by malignant cells.^2,3^

The cornerstone of treatment for upper tibial chondroblastic osteosarcoma is multimodal therapy, which includes neoadjuvant chemotherapy, surgical resection, and adjuvant therapy.^1,2^ Neoadjuvant chemotherapy seeks to shrink the tumor, reduce its size, and eliminate micrometastatic illness.^3^ Surgical excision with wide margins is required to achieve local control and reduce the chance of recurrence. Adjuvant chemotherapy can be given postoperatively to target residual illness and avoid distant metastases.^4,5^

Despite breakthroughs in multimodal therapy, the prognosis for chondroblastic osteosarcoma remains uncertain, especially in cases of advanced disease at presentation or insufficient surgical resection margins.^2^ The overall survival rate varies according to tumor stage, histological grade, treatment response, and the occurrence of metastases.^4^ Long-term follow-up is required to check for cancer recurrence and late treatment effects, such as the development of second malignancies or treatment-related problems.^1,5^

More studies are needed to determine the molecular pathways behind chondroblastic osteosarcoma carcinogenesis and progression.^3,4^ This could help develop targeted therapy tailored to the precise genetic abnormalities and signaling pathways involved in the disease. Furthermore, prospective research examining novel treatment techniques and predictive biomarkers is needed to increase therapy efficacy and patient outcomes.^1,2^

Chondroblastic osteosarcoma of the upper tibia is challenging to diagnose and treat because of its rarity and anatomical position.^4^ A multidisciplinary strategy that includes precise diagnosis, neoadjuvant chemotherapy, surgical resection, and adjuvant therapy is critical for optimizing results in these patients.^1^ Long-term follow-up is required to check for illness recurrences and provide timely intervention if warranted. This example highlights the significance of comprehensive management measures in the treatment of chondroblastic osteosarcoma.^4,5^

**Figure 1:**
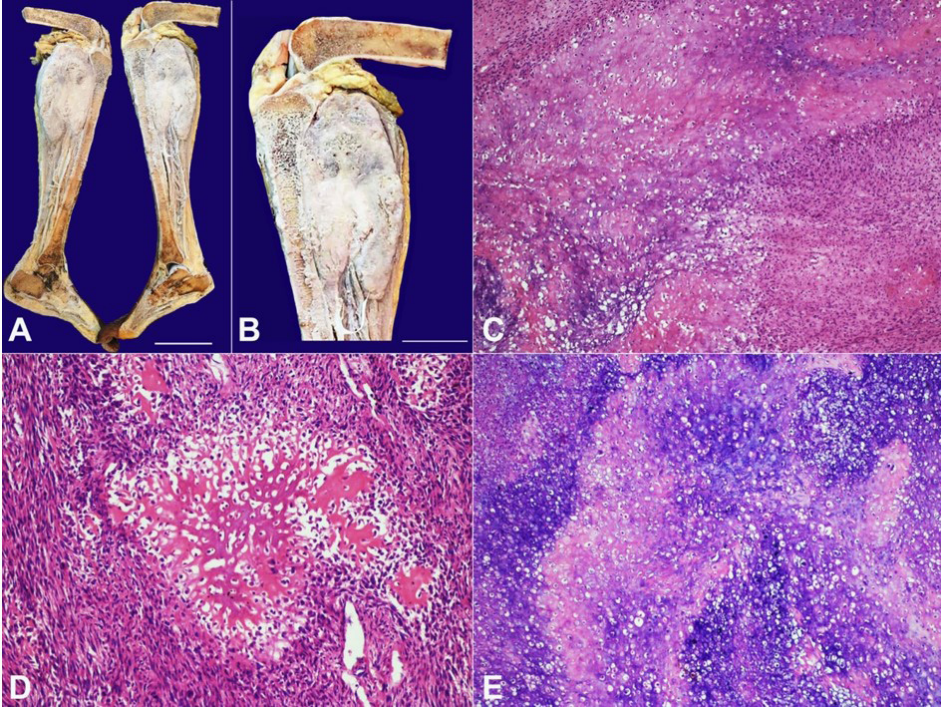
**A –** Gross view of the above knee amputation showing a greyish faint white tumor in the proximal tibia (scale bar = 14 cm); **B –** shows a grey-white infiltrative tumor involving the upper end of the proximal tibia (scale bar = 10 cm), C - shows islands of osteoid matrix within the cartilaginous area. The periphery of the cartilaginous areas shows condensation and spindling of the tumor cells (H&E x100); **C –** shows areas of osteoid production with spindling of the tumor cells (H&E x200); **D –** shows cartilaginous differentiation of the tumor with foci of osteoid production in this chondroblastic osteosarcoma (H&E x200).

Figure 1 refers to a 13-year-old female patient who presented to our hospital complaining of pain and swelling in the right knee region for three months. There was no history of trauma, weight loss, or anorexia. On inspection, the right knee showed diffuse swelling of 20 x 20 cm, with no engorged veins, open wound, or discharge. On palpation, there was a local rise in temperature with severe tenderness, hard consistency, and diffuse margin in the right knee. The X-ray was consistent with a lytic sclerotic epiphysial-metaphyseal lesion of the proximal tibia, which had a large soft tissue component breaching the cortex. A computed tomography angiogram (CT angiogram) shows a heterogeneously enhancing expansile lytic destructive lesion with aggressive periosteal reaction seen over the meta-diaphyseal region of the upper end of the right tibia reaching up to epiphysis and involving joints. A large heterogeneously enhancing lobulated soft tissue component with osteoid matrix and calcific foci measuring 11 x 13 x 14 cm seen reaching up to fascia was depicted post-contrast. Contrast-Enhanced Magnetic Resonance Imaging (CEMRI) was consistent with an aggressive lesion involving the proximal epi-metaphyseal region of the right tibia with intra-articular extension and invasion to adjacent structures. A biopsy of the proximal tibial lesion showed features of dedifferentiated osteosarcoma. Subsequently, the patient was operated on for the above-knee amputation without any complications. Microscopic examination of the proximal tibial lesion shows a cellular tumor infiltrating bone and soft tissue. Tumor cells exhibited moderate to marked pleomorphism, including vesicular nuclei, prominent nucleoli, and moderate cytoplasm. Lacy-like osteoid material was seen between tumor cells. There were frequent mitoses but no necrosis. The cartilage nests and lobules showed enhanced cellularity, nuclear atypia, and osteoid matrix material revealed histomorphological features of Chondroblastic osteosarcoma. The tumor did not involve all the resection margins. The patient had an uneventful postoperative recovery. The patient was scheduled for regular follow-up to monitor disease progression with imaging studies to detect any local recurrence or metastasis signs.
